# *DPYD*-guided fluoropyrimidine dose adjustment in colorectal cancer *DPYD* carriers: start slower to finish stronger

**DOI:** 10.3389/fphar.2025.1645188

**Published:** 2025-09-18

**Authors:** Rocío Rosas-Alonso, Nuria Rodríguez Salas, Pablo Perez Wert, Angela Hoyo, Susana Martin-López, Daniel Martínez-Pérez, Iciar Ruiz-Gutiérrez, Diego Jiménez-Bou, Jesús Peña, Pedro Arias, Ana Custodio, Itsaso Losantos-García, Alberto M. Borobia, Jaime Feliu, Ismael Ghanem

**Affiliations:** ^1^ Biomarkers and Experimental Therapeutics in Cancer, La Paz University Hospital Research Institute (IdiPAZ), Madrid, Spain; ^2^ Pharmacogenetic Unit, La Paz University Hospital, Madrid, Spain; ^3^ Translational Oncology, La Paz University Hospital Research Institute (IdiPAZ), Madrid, Spain; ^4^ Medical Oncology Department, La Paz University Hospital, Madrid, Spain; ^5^ Pharmacy Department, La Paz University Hospital, Madrid, Spain; ^6^ Biostatistics Platform. La Paz University Hospital Research Institute (IdiPAZ), Madrid, Spain; ^7^ Clinical Pharmacology, La Paz University Hospital Research Institute (IdiPAZ), Madrid, Spain; ^8^ Biomedical Research Networking Center on Oncology-CIBERONC, ISCIII, Madrid, Spain

**Keywords:** fluoropyrimidines, DPYD, colorectal cancer, toxicity, discontinuation

## Abstract

**Introduction:**

Fluoropyrimidines (FP) are the mainstay of colorectal cancer (CRC) treatment, but can cause severe toxicity in up to 40% of patients. Variants in the *DPYD* gene are associated with these adverse events. A *DPYD*-guide dose adjustment is now recommended in Europe. This ambispective study aims to analyze the FP-related severe toxicity and the FP dose adjustment in heterozygous *DPYD* variant carriers with colorectal cancer, comparing a *DPYD*-guided FP dose adjustment (DA) approach to the non-*DPYD*-guided FP dose adjustment (NDA).

**Methods:**

1.279 *DPYD* genotyping reports were issued. Sixty patients were identified with *DPYD* variants. Twenty-five CRC patients (17 in the DA cohort and 8 in the NDA cohort) were included in the analysis. Thirty-five patients were excluded from the analysis because they did not satisfy any of the study’s inclusion criteria. Reasons for exclusion included having a diagnosis other than colorectal cancer, not receiving fluoropyrimidine treatment, participation in a clinical trial, or insufficient follow-up.

**Results:**

Of the twenty-five patients included, sixteen patients (94%) started with a 50% FP dose reduction in the DA cohort while 7 out of 8 patients (87%) in the NDA cohort received 100% of dose in cycle 1. In the DA cohort, 12% of patients experienced severe fluoropyrimidines-related adverse events, compared to 50% in the NDA cohort (OR = 0.13; 95%CI: 0.01–0.93; p = 0.05). FP discontinuation due to severe toxicity occurred in 6% of patients in the DA cohort versus 50% in the NDA cohort (OR = 0.06; 95%CI: 0.003–0.55, p = 0.02).

**Discussion:**

These findings suggest that *DPYD*-guided dose adjustment significantly reduces both the incidence of severe toxicity and the rate of treatment discontinuation in CRC patients. Initiating treatment with a 50% FP reduction allows for dose escalation in patients who exhibit good tolerance and avoid the discontinuation for those patients intolerant to higher doses thereby improving overall treatment adherence and completion.

## 1 Introduction

Colorectal cancer (CRC) ranks as the third most common neoplasm globally and stands as the second leading cause of cancer-related mortality (Bray et al., 2024). Chemotherapy based on fluoropyrimidine (FP) is widely utilized for various tumor types, forming a cornerstone in the treatment of gastrointestinal tumors, particularly CRC. FP can be administered as monotherapy or in combination with other agents such as platinum compounds, irinotecan, taxanes, or monoclonal antibodies (Cervantes et al., 2023; Argiles et al., 2020).

Severe toxicity is observed in up to 40% of patients undergoing FP chemotherapy, with mortality rates from adverse events ranging from 0.5% to 1% (Levy et al., 1998; Hoff et al., 2001). Certain clinical characteristics such as an older age, or female sex may be associated with a higher risk of severe toxicity (Knikman et al., 2021). In addition, the critical enzyme in the metabolism and inactivation of FP is dihydropyrimidine dehydrogenase (DPD). Variants in the *DPYD* gene, which encodes DPD, are key determinants of reduced enzyme activity. A deficiency in DPD function is present in 39%–61% of patients experiencing severe FP toxicity (van Kuilenburg, 2004). Therefore, *DPYD* variants significantly increase the risk of severe toxicity at standard doses (Rosmarin et al., 2014; Ruzzo et al., 2017; Meulendijks et al., 2015; Henricks et al., 2018). However, a large recent meta-analysis reported that among patients developing grade 3–4-5 hematologic and digestive fluoropyrimidine-related toxicities (no dose adjustment on *DPYD*), only 14% and 7% carried at least one *2A, *13 or c.2846A>T allele, or c.1236G>A (haplotype B3) allele, respectively (Le et al., 2024).

In a prospective study, Henricks, L. M. et al. Demonstrated that an initial 50% dose reduction of FP may be sufficient for heterozygous carriers of the nonfunctional DPYD*2A and DPYD*13 variants. However, due to the low prevalence of these variants, the study included only 16 patients heterozygous for DPYD*2A and 1 patient heterozygous for DPYD*13. For variants associated with reduced function (c.2846A>T and c.1236G>A), a 25% dose reduction was considered potentially insufficient, necessitating further investigation (Henricks et al., 2018).

Based on the findings of increased risk of severe toxicity with these variants (Rosmarin et al., 2014; Meulendijks et al., 2015; Terrazzino et al., 2013), several clinical guidelines recommend performing *DPYD* genotyping analysis in all patients prior to starting FP treatment and adjusting the dose if a deficiency is detected (Amstutz et al., 2018; Lunenburg et al., 2020; Garcia-Alfonso et al., 2022). However, these guidelines differ in their recommendations for initial dose adjustments and do not provide detailed guidance on increasing FP doses based on specific *DPYD* variants.

Furthermore, numerous studies have shown that *DPYD* variant carriers receiving standard treatment have a higher risk of severe adverse events compared to the wild-type population (Rosmarin et al., 2014; Ruzzo et al., 2017), even after genotype-guided dose adjustments (Henricks et al., 2018; Deenen et al., 2016). However, there are no studies directly comparing the benefits in terms of toxicity of FP treatment in *DPYD* carries with or without *DPYD*-guided dose adjustments according to current recommendations, specifically in CRC patients.

Consequently, additional research is imperative to elucidate the clinical management of unresolved questions concerning *DPYD*-guided FP dose adjustments.

The aim of our study is to analyze the FP toxicity profile in routine clinical practice, as well as the dose FP adjustment dynamics in heterozygous patients carrying any of the four classical *DPYD* variants, depending on whether they received a *DPYD*-guided FP dose adjustment or no adjustment.

## 2 Materials and methods

### 2.1 Study population

The inclusion criteria of the study were: patients diagnosed with colorectal cancer (CRC) at Hospital Universitario La Paz; identification of a *DPYD* gene variant by genotyping between 1 June 2020, and 28 June 2024; initiation of first-time treatment with fluoropyrimidines (FP), either before or after *DPYD* genotyping, administered as monotherapy or in combination with oxaliplatin (with or without targeted therapies); treatment given in routine clinical practice as (neo)adjuvant therapy or first-line therapy for metastatic disease; to have provided informed consent for pharmacogenetic testing.

The exclusion criteria were: participation in a clinical trial; concurrent treatment with irinotecan; diagnosis of a non-colorectal malignancy; and a follow-up duration of less than 3 months.

### 2.2 Study design

This is a single-center, ambispective study conducted at Hospital Universitario La Paz. The study was conducted with the approval of the Ethics Committee of La Paz University Hospital (PI-5204) and in accordance with the principles outlined in the Declaration of Helsinki. Two cohorts were established: the *DPYD*-guided FP dose adjustment cohort (DA), consisting of patients who received a FP dose adjustment in cycle 1 for *DPYD* variant carriers, with subsequent dose escalation based on patient tolerance; and the non-*DPYD*-guided FP dose adjustment cohort (NDA), comprising patients who received an unadjusted *DPYD*-guide FP dose, higher than 75%, in the first cycle. Most of the patients in the NDA cohort were treated prior to the implementation of preemptive *DPYD* genotyping in our hospital (June 2020). These patients were subsequently genotyped due to relapse or second malignancy.

Clinical and genetic variables were collected. Clinical variables were extracted from patient medical records, encompassing a comprehensive array of data. These included sex, age, ECOG performance status, therapeutic intention and treatment regimen administered. Severe (≥ grade 3) FP-related adverse events (SFAEs) were evaluated according to the Common Terminology Criteria for Adverse Events (CTCAE) version 5.0 at cycle 1 and maximum toxicity at any cycle. Maximum FP dose, initial and final dose adjustments, and discontinuation of FP due to toxicity were also collected. To minimize bias from overestimating FP-related toxicity in patients receiving combination regimens, patients treated with irinotecan (associated with overlapping toxicities) were excluded, and only adverse events specifically attributable to FP were considered. FP-related adverse events were defined as follows: diarrhea, fatigue, emesis, hand-foot syndrome, neutropenia, thrombopenia, anemia, febrile neutropenia, mucositis and hyperbilirubinemia.

### 2.3 DNA extraction and genotyping

The DNA extraction and genotyping were performed during routine clinical practice in the Clinical Pharmacogenetics Unit, a multidisciplinary unit integrating the clinical pharmacology and genetics departments (Stewart et al., 2023; Borobia et al., 2018). For the genetic study, blood samples were collected in Vacutainer tubes containing EDTA anticoagulant (Becton Dickinson, Franklin Lakes, NJ, United States). DNA was then isolated in the preanalytical unit of the genetics department using the Chemagen robot (Perkin-Elmer, Boston, MA, United States). *DPYD* genotyping was performed in the pharmacogenetics laboratory using a validated OpenArray technology (ThermoFisher, Waltham, MA, United States) (Rosas-Alonso et al., 2021). Specifically, for the pharmacogenetics of FP, the four variants recommended by the Spanish Agency for Medicines and Medical Devices (AEMPS) were analyzed in a clinical setting: DPYD*2A (rs3918290, c.1905 + 1G>A), DPYD*13 (rs55886062, c.1679T>G), c.2846A>T (rs67376798) and [c.1236G>A; c.1129–5923C>G]/HapB3) (rs56038477 and rs75017182). Both HapB3 haplotype variants were analyzed to confirm linkage disequilibrium.

### 2.4 Treatment recommendations

Based on the genotype of both alleles for these variants, an activity score was assigned with a dose reduction recommendation according to the Clinical Pharmacogenetics Implementation Consortium (CPIC) guidelines (Amstutz et al., 2018). Thus, heterozygous c.2846A>T and c.1236G>A carriers (reduced function variants) were assigned an activity score of 1.5 and dose reductions of 25%–50% were recommended, depending on individual patient conditions (and oncologist decision). If the patient showed good clinical tolerance, in subsequent cycles (tipically the second), the dose was usually increased up to 75% at the discretion of the treating physician. For heterozygous DPYD*2A or DPYD*13 carriers (loss of function variants), an activity score of 1.0 was assigned and a dose reduction of 50% was recommended. No dose escalation was commonly performed in subsequent cycles even when clinical tolerance was adequate. However, doses could be adjusted according to individual patient circumstances and medical decisions. For the compound heterozygous carrier with an activity score of 1, the CPIC guideline does not provide specific recommendations; therefore, our recommendations were based on the DPWG guideline (Lunenburg et al., 2020), specifically, an additional phenotyping test measuring uracil levels to determine DPD enzyme activity was performed, as dose adjustments cannot be accurately predicted based on genotype alone according to the DPWG guideline. Relative dose intensity was calculated as the dose administered divided by the standard dose for the regimen administered.

### 2.5 Statistical analysis

Qualitative variables were presented as absolute frequencies and percentages. Quantitative variables were summarized using means or medians, depending on their distribution. The normality of continuous variables was assessed using the Shapiro-Wilk test. Associations between categorical variables were analyzed using binary logistic regression (severe toxicity and treatment discontinuation), Fisher’s test (qualitative baseline characteristics) and t-Student (age). All statistical tests were two-sided, with p-values ≤0.05 considered statistically significant. Data analysis was performed using R statistical software, version 4.3.3 (de With et al., 2023).

## 3 Results

### 3.1 Characteristics of patients

**FIGURE 1:**
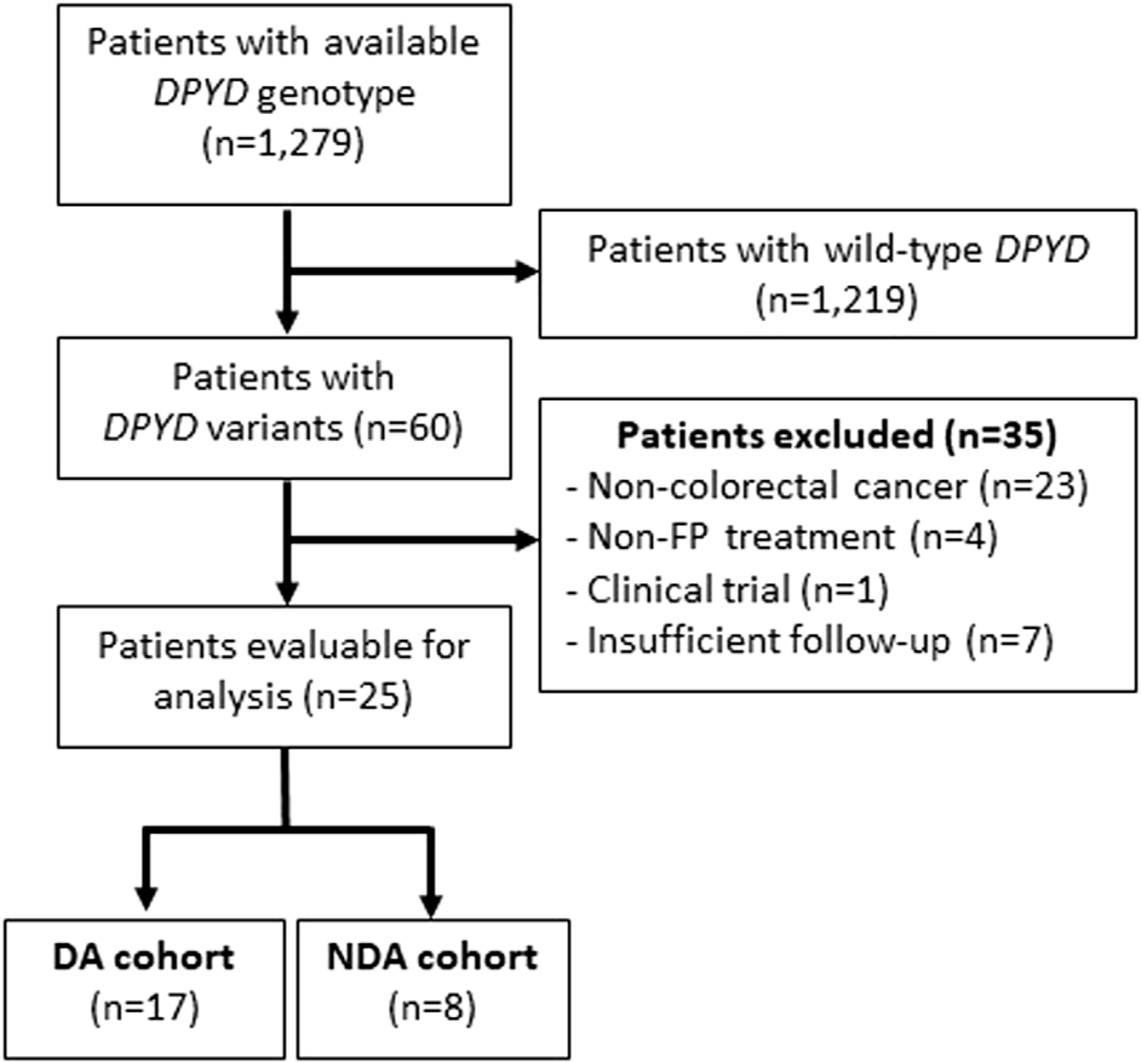
Flow diagram for the inclusion of patients D*PYD*: dihydropyrimidine dehydrogenase; FP: Fluoropyrimidines; AD: *DPYD*-guided FP dose adjustment; NDA: Non-*DPYD*-guided FP dose adjustment.

From 1 June 2020 to 28 June 2024 a total of 1,279 *DPYD* genotyping reports were issued. Among these, 60 patients (4.7%) were identified with *DPYD* variants (Supplementary Table S1). Specifically, 25 of these *DPYD*-variant carriers were diagnosed with CRC and received FP based chemotherapy. Thirty-five patients were excluded from the analysis because they did not meet any of the study inclusion criteria. Seventeen patients (68%) were included in the DA cohort while 8 were included in the NDA cohort (Figure 1).

The baseline characteristics of the patients are presented in Table 1, showing a generally well-balanced distribution between the two cohorts, with the exception of sex (65% female in the DA cohort compared to 12% in the NDA cohort). Most patients exhibited an ECOG performance status of 0–1, with 88% in the DA cohort and 100% in the NDA cohort. All patients in the study received chemotherapy regimens consisting of FP, either as monotherapy or in combination with oxaliplatin. Additionally, six patients (24%) were treated with bevacizumab or anti-EGFR antibodies, and only one patient received concurrent radiotherapy with capecitabine. More than half of the patients carried the c.1236G>A variant, with prevalence rates of 65% and 50% in the DA and NDA cohorts, respectively. There was one patient who was compound heterozygous for both c.1236G>A and c.2846A>T.

**TABLE 1:** Baseline characteristics of patients in the DA and NDA cohorts.

Characteristic	Overall population (n = 25)	DA cohort (n = 17)	NDA cohort (n = 8)	p-value
Age				
Years, mean (min-max)	70 (46–90)	68 (46–90)	73 (61–86)	0.37
Sex				
Male	13 (52%)	6 (35%)	7 (88%)	0.03
Female	12 (48%)	11 (65%)	1 (12%)	
ECOG				
0–1	23 (92%)	15 (88%)	8 (100%)	1.00
2	2 (8%)	2 (12%)	0 (0%)	
Treatment regimen				
Capecitabine	6 (24%)	4 (24%)	2 (25%)	0.75
XELOX	12 (48%)	8 (47%)	4 (50%)	
Capecitabine plus radiotherapy	1 (4%)	0 (0%)	1 (12%)	
Capecitabine plus Bevacizumab	1 (4%)	1 (5%)	0 (0%)	
FOLFOX/XELOX plus mab*	5 (20%)	4 (24%)	1 (13%)	
Treatment intention				
Adjuvant	16 (64%)	10 (59%)	6 (75%)	0.66
Fist-line	9 (36%)	7 (41%)	2 (25%)	
Stage				
II	5 (20%)	3 (18%)	2 (25%)	0.97
III	11 (44%)	7 (41%)	4 (50%)	
IV	9 (36%)	7 (41%)	2 (25%)	
*KRAS* status				
Mutated	9 (69%)	5 (71%)	4 (67%)	1.00
No mutated	4 (31%)	2 (29%)	2 (33%)	
Not available	12	10	2	
*BRAF* status				
Mutated	1 (7%)	1 (11%)	0 (0%)	1.00
No mutated	14 (93%)	8 (89%)	6 (100%)	
Not available	10	8	2	
MMRd status				
Yes	2 (8%)	2 (12%)	0 (0%)	1.00
No	21 (84%)	14 (82%)	7 (88%)	
Not available	2	1	1	
*DPYD* status				
c.1236G>A heterozygous<	15 (60%)	11 (65%)	4 (50%)	0.67
c.2846A>T heterozygous	5 (20%)	3 (17%)	2 (25%)	
DPYD*2A heterozygous	3 (12%)	1 (6%)	2 (25%)	
DPYD*13 heterozygous	1 (4%)	1 (6%)	0 (0%)	
c.1236G>A and c.2846A>T heterozygous	1 (4%)	1 (6%)	0 (0%)	

mab*: Monoclonal antibody including: Bevacizumab, cetuximab or panitumumab; AD: *DPYD*-guided FP dose adjustment; NDA: Non-*DPYD*-guided FP dose adjustment. The p-value was calculated using Fisher’s exact test for qualitative variables. For age, normality was confirmed using the Shapiro-Wilk test (p = 0.06), and the p-value for age was calculated using Student’s t-test.

### 3.2 Severe fluoropyrimidine related toxicity

**FIGURE 2:**
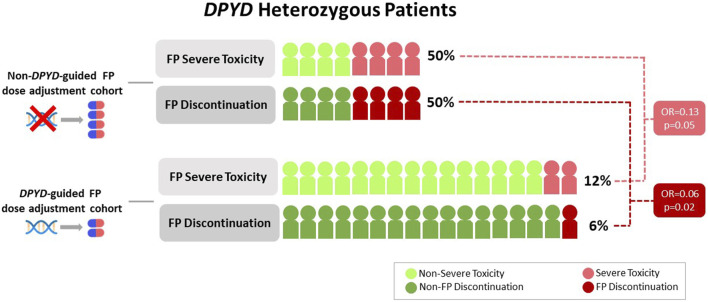
Severe fluoropyrimidine-related toxicity and treatment discontinuation in DA and NDA cohorts.

Overall, 6 patients (24%) experienced SFAEs, 2 patients (12%) from the DA cohort and 4 patients (50%) from the NDA cohort (OR = 0.13; 95%CI: 0.01–0.93; p = 0.05) (Figure 2). Specifically, regarding the most frequent variant (c.1236G>A), there were no SFAEs in patients within the DA cohort, while 50% in the NDA cohort experienced SFAEs (Table 2). In the DA cohort, 6% of the patients were hospitalized due to treatment-related toxicities, whereas in the NDA cohort, the rate was 25%.

**TABLE 2:** Severe (≥ grade 3) FP-related adverse events in DA and NDA cohorts (n ≥ 1).

SFAEs	Overall population (n = 25)	DA cohort (n = 17)	NDA cohort (n = 8)
Overall SFAEs	6 (24%)	2 (12%)	4 (50%)
Diarrhea			
Overall	4 (16%)	1 (6%)	3 (37%)
Cycle 1	2 (8%)	1 (6%)	1 (12%)
Neutropenia			
Overall	2 (8%)	1 (6%)	1 (12%)
Cycle 1	0 (0%)	0 (0%)	0 (0%)
Fatigue			
Overall	2 (8%)	1 (6%)	1 (12%)
Cycle 1	0 (0%)	0 (0%)	0 (0%)
Hyperbilirubinemia			
Overall	1 (4%)	0 (0%)	1 (12%)
Cycle 1	0 (0%)	0 (0%)	0 (0%)
Emesis			
Overall	1 (4%)	0 (0%)	1 (12%)
Cycle 1	0 (0%)	0 (0%)	0 (0%)
Fluoropyrimidine-related hospital admission	3 (12%)	1 (6%)	2 (25%)

SFAEs, Severe (≥grade 3) FP-related adverse events; DA, *DPYD*-guided FP dose adjustment; NDA, Non-*DPYD*-guided FP dose adjustment.

The most frequent SFAEs was diarrhea in 4 patients (16%). Other SFAEs were neutropenia (8%), fatigue (8%), hyperbilirubinemia (4%) and emesis (4%). All them were most frequent in the NDA cohort (Table 2). There were no deaths related to the FP administration.

The SFAEs according to the type of DPYD variant are presented in Table 3.

**TABLE 3:** Severe (≥ grade 3) FP-related adverse events in DA and NDA cohorts according to *DPYD* variants.

SFAEs	Overall population (n = 25)	DA cohort (n = 17)	NDA cohort (n = 8)
c.1236G>A (n = 15)	2 (14%)	0 (0%)	2 (50%)
c.2846A>T (n = 5)	2 (40%)	1 (33%)	1 (50%)
DPYD*2A (n = 3)	2 (67%)	1 (100%)	1 (50%)
DPYD*13 (n = 1)	0 (0%)	0 (0%)	—
c.2846A>T+c.1236G>A (n = 1)	0 (0%)	0 (0%)	—

SFAEs, Severe (≥grade 3) FP-related adverse events; DA, *DPYD*-guided FP dose adjustment; NDA, Non-*DPYD*-guided FP dose adjustment.

### 3.3 Fluoropyrimidine dose adjustment dynamics

Due to SFAEs, FP treatment was adjusted in 7 out of 8 patients (87%) in the NDA cohort. In the DA cohort, dose reduction was performed in only one case (6%), whereas for the remaining patients, the doses were either maintained or increased.

The FP dosing and modifications or dynamics of dose adjustments for the DA and NDA cohorts are summarized in Table 4. A normality test was conducted for the variables initial dose, maximum dose, and final dose using the Shapiro-Wilk test. In all cases, the p-value was <0.05, indicating that the data did not follow a normal distribution. In the DA cohort, the median relative dose intensity for the initial dose was 50%. Notably, 94% of patients received 50% of the standard dose, while one patient received a higher dose (62%). In the NDA cohort 7 out of 8 patients (87%) received 100% of standard dose in cycle 1. Regarding the maximum dose, the DA cohort had a median relative dose intensity of 70%, with 76% of patients receiving doses ranging from 50% to 75%. The NDA cohort had a median relative dose intensity of 100%, with 88% of patients receiving 100%.

**TABLE 4:** Fluoropyrimidine dose adjustment dynamics according to the DA and NDA cohorts.

	DA (n = 17)	NDA (n = 8)
Initial dose		
Relative dose intenasity*, median (min-max)	50%; (50–62)	100%; (80–100)
Dose 50%, n (%)	16 (94%)	0 (0%)
Dose >50%–75%, n (%)	1 (6%)	0 (0%)
Dose >75%, n (%)	0 (0%)	8 (100%)
Maximum dose		
Relative dose intensity*, median (min-max)	70%; (50–90)	100%; (80–100)
Dose 50%, n (%)	2 (12%)	0 (0%)
Dose >50%–75%, n (%)	13 (76%)	0 (0%)
Dose >75% - <100%, n (%)	2 (12%)	1 (12%)
Dose 100%, n (%)	0 (0%)	7 (88%)
Final dose		
Relative dose intensity*, median (min-max)	70%; (0–90)	25%; (0–100)
Dose 0%–50% **, n (%)	3 (18%)	5 (63%)
Dose >50%–75%, n (%)	12 (70%)	1 (12%)
Dose >75%, n (%)	2 (12%)	2 (25%)

*Relative dose intensity: the given dose relative to the standard dose. **If the treatment was discontinued, the final dose was considered 0%. FP, Fluoropyrimidines; DA, *DPYD*-guided FP dose adjustment cohort; NDA, No *DPYD*-guided FP dose adjustment cohort.

For the final dose, the DA cohort had a median relative dose intensity of 70%, with 70% of patients receiving more than 50%–75%. The NDA cohort had a median relative dose intensity of 25%, with 63% of patients receiving 0%–50%.

FP discontinuation due to toxicity occurred in 6% of patients in the DA cohort and 50% of patients in the NDA cohort (OR = 0.06; 95%CI; 0.003–0.55, p = 0.02) (Figure 2). In the DA cohort, treatment discontinuation due to toxicity occurred after the third cycle. In the NDA cohort, treatment discontinuation occurred after the first, the second, the third and the fourth cycles.

In the subgroup of patients with decreased function *DPYD* variants, the initial dose in the DA cohort had a median relative dose intensity of 50%, with all patients receiving 50% of the dose. The NDA cohort had a median initial dose intensity of 100%, with all patients receiving doses greater than 75%. At the final dose, the DA cohort had a median intensity of 75%, whereas the NDA cohort had a median relative dose intensity of 37% (Supplementary Table S2). Additionally, 50% of the NDA cohort discontinued FP due to toxicity, compared to none in the DA cohort (p = 0.99).

Only four patients had *DPYD* variants with loss of function, two in the NDA cohort and two in the DA cohort. In the NDA cohort, both patients were heterozygous for the DPYD*2A variant. One patient discontinued treatment at cycle 2 after experiencing grade 3 diarrhea and grade 3 neutropenia, while the other patient presented with grade 2 hematological toxicity, requiring a 50% dose reduction to complete the treatment. In the DA cohort, one DPYD*2A carrier discontinued FP at the third cycle after experiencing grade 3 diarrhea, while the other patient, a DPYD*13 carrier, completed the treatment without severe adverse effects.

Finally, the compound heterozygous carrier for c.2846A>T and c.1236G>A completed the FP treatment, starting with a 50% dose reduction, increasing the dose to 66%. In this case, both genotyping and phenotyping were performed: while the genotype inferred an activity score of 1 or partial DPD deficiency, the phenotyping result indicated normal DPD activity. Given this discrepancy, we opted for the safest approach, managing the patient as an activity score 1 carrier.

## 4 Discussion

The implementation of pharmacogenetic testing for fluoropyrimidines has become a reality in Europe following the European Medicines Agency and European Society of Medical Oncology to recommend preemptive *DPYD* testing (de With et al., 2023; Abad-Santos et al., 2024). However, there remain unresolved issues regarding optimal dose adjustment and dose escalation.

Our study showed a prevalence of 4.7% of carriers, which is lower than the frequencies reported in other studies, which reached 8% (Henricks et al., 2018). However, this discrepancy may be due to a lower frequency of these variants in the Spanish population, with previous reports indicating frequencies below 5% (Miarons et al., 2023).

In our study, patients were treated using either a *DPYD*-guided FP dose adjustment strategy or a standard dose FP-based treatment. In the DA cohort, patients started with a median FP dose intensity of 50% (n = 17), whereas the NDA cohort received 100% (n = 8). Consequently, patients in the DA cohort experienced fewer SFAEs and were less likely to discontinue treatment.

Remarkably, the rate of severe toxicity in the NDA cohort (50%) was four times higher than that observed in the DA cohort (12%). A recently published study involving 27 patients, but not limited to CRC, also shows that *DPYD* genotype pretreatment testing reduced severe toxicities in *DPYD* variant carriers from 64% to 31% (Nguyen et al., 2024). The rate of severe toxicity in the NDA cohort was similar to that reported in previous studies (Rosmarin et al., 2014; Meulendijks et al., 2015; Terrazzino et al., 2013; Nguyen et al., 2024). However, the incidence of SFAEs for the DA cohort was substantially inferior to the 39% reported in the prospective Henricks, L. M. et al. Study, also evaluating the *DPYD* genotype-guided FP dose adjustment strategy (Henricks et al., 2018). These differences could be explained by the variability secondary to the low incidence of *DPYD* variants, the partially retrospective data collection that could underreport the toxicity in this work or the homogeneity and less toxic regimens and neoplasms in our study. However, the larger 50% dose reduction performed in the first cycle for decreased function *DPYD* variants in this study compared to 25% in the Henricks study seems the most consistent explanation. The overall dose escalation rate of 13% in the Dutch study does not justify its higher toxicity (Henricks et al., 2018). Other studies starting on a 50% dose reduction also showed lower severe toxicity rates of 20%–30% (Nguyen et al., 2024; Wang et al., 2022). Although there is a significantly higher proportion of males in the NDA cohort (p = 0.03), this fact does not seem to justify the differences in severe toxicity, since it is the female sex that could potentially be associated with a higher risk of toxicity (Knikman et al., 2021).

Another important question is whether the *DPYD*-guided reduction of FP doses could affect the prognosis of oncologic diseases, particularly in CRC. The Knikman study suggests that while no apparent loss of efficacy was observed in the overall pool of patients carrying *DPYD* variants, a loss of efficacy in carriers of the c.1236G>A variant compared to *DPYD* wild-type patients cannot be ruled out (Knikman et al., 2023). In our study, survival was not evaluated due to a potential selection bias, as the vast majority of patients in the NDA cohort were identified by *DPYD* analysis after relapse. However, when comparing the dynamics of FP dose administration in both cohorts, 50% of patients carrying *DPYD* variants treated without dose adjustment discontinued FP in the first cycles, whereas only 6% of patients following *DPYD*-guided FP dose adjustment had to discontinue FP due to SFAEs. This discontinuation rate is even lower than that reported for the wild-type *DPYD* population or for *DPYD* variant carriers treated with a more permissive FP dose reduction strategy, which showed discontinuation rates above 18% (Henricks et al., 2018). Additionally, in the DA cohort, the majority (82%) of patients received a dose greater than 50% in the last cycle, with a median relative dose intensity of 70%.

Therefore, the data from our study which exclusively involved CRC patients support that overall, the *DPYD*-guided dose adjustment strategy not only reduces the risk of toxicity but also reduces the risk of FP discontinuation, making it unlikely to result in a worse prognosis.

However, other questions remain open: how much to adjust the FP dose in the first cycle, whether dose escalation is indicated, or the rate of dose escalation for each *DPYD* variant to minimize the risk of toxicity while maintaining the maximum efficacy of FP (Henricks et al., 2018; Amstutz et al., 2018; Lunenburg et al., 2020; Knikman et al., 2023). Focusing on the *DPYD* variants with reduced function (c.2846A>T and c.1236G>A), our study suggests that initiating treatment with a 50% dose reduction, followed by an escalation up to 75% based on clinical tolerance, results in a marked decrease in the risk of severe toxicity, from 50% to less than 10%. This approach addresses the issue of insufficient decrease in the incidence of severe toxicity with an initial 25% dose reduction shown in the largest prospective study (Henricks et al., 2018). Furthermore, with this approach no patients had to discontinue FP treatment.

This is of particular interest when considering the prognostic uncertainty for patients with the c.1236G>A variant who were treated with an initial 75% dose of FP (Knikman et al., 2023). On the other hand, a less aggressive approach, starting at 50% of the standard dose without dose escalation could not add any benefit in tolerance and potentially lead to under-treatment for most patients (Wang et al., 2022). Our study also suggests the possibility of exploring a dose escalation beyond 75% for carriers of reduced function variants: 93% of patients were able to complete the treatment with a dose escalation at least up to 75%. A small study with 11 patients showed that a tolerance-based capecitabine dose escalation was possible in 4 patients (Kleinjan et al., 2019). However, in the Dutch study, three out of five patients with *DPYD* variants with reduced function had to reduce the FP dose after a dose escalation beyond 75% (Henricks et al., 2018).

Concerning *DPYD* variants with loss of function, the evidence is much more limited. The results of our study in only 4 patients (3 for DPYD*2A and 1 for c.1679T>G) showed that one patient in each cohort discontinued the treatment due to severe toxicity, regardless of the 50% dose adjustment. However, the largest prospective study evaluating the dose adjustment in DPYD*2A carriers (n = 22) showed that an initial 50% dose reduction was safe. In 3 patients (17%) a further reduction was necessary because of toxicity (Deenen et al., 2016). Another study with 17 *DPYD* variant carriers (16 for variant DPYD*2A and 1 for c.1679T>G) suggests that a 50% reduction is safe as well (Henricks et al., 2018).

Therefore, a treatment strategy that begins with lower doses followed by careful dose escalation could potentially identify patients with poor tolerance, preventing severe toxicity and ensuring treatment completion. Additionally, DPD may be an inducible enzyme (Yoshida et al., 2015), which could allow for higher doses to be reached with good tolerance. Hence, for heterozygous patients with loss-of-function *DPYD* variants an initial dose reduction of ≥50%, with subsequent dose escalation based on patient tolerance up to 50%. For heterozygous patients with reduced-function *DPYD* variants an initial dose reduction of 50%, followed by dose escalation based on patient tolerance up to 75%. While a higher limit may be tolerable for some patients, we believe that further research is necessary.

These proposed strategies are consistent with international pharmacogenetic recommendations, although some variability exists across clinical guidelines. The Spanish Pharmacogenetics and Pharmacogenomics Society, together with the Spanish Society of Medical Oncology, recommend initiating treatment at 50% of the standard dose, followed by dose titration according to toxicity or pharmacokinetic parameters (Garcia-Alfonso et al., 2022). Likewise, the DPWG advises starting therapy at 50% of the standard dose for patients with an activity score of 1 or 1.5, with subsequent adjustments based on observed toxicity and therapeutic effectiveness (Lunenburg et al., 2020). Both approaches are closely aligned with the strategy we propose. By comparison, the French National Network of Pharmacogenetics and the Italian Association of Medical Oncology propose a somewhat less cautious approach, recommending a 50% dose reduction for heterozygous carriers of *2A, *13, or c.2846A>T variants during the first cycle, while for carriers of c.1236G>A, initiation at 75% of the standard dose is advised (Quaranta and Thomas, 2017). Finally, the CPIC recommends a 50% dose reduction for activity score 1 carriers, whereas for those with an activity score of 1.5 the guidance is less specific, suggesting a reduction of between 25% and 50% depending on clinical circumstances.

This study has several limitations as a single-center ambispective study, potentially leading to underestimated data collection, particularly regarding toxicity. Additionally, the small sample size restricts the ability to draw definitive conclusions. Furthermore, we cannot draw conclusions about efficacy due to potential selection bias. In addition, the fact that the NDA cohort consisted primarily of patients with recurrent or secondary tumors could potentially influence the toxicity results. Nevertheless, the low prevalence of patients with *DPYD* variants underscores the significance of this study, as it addresses questions that had not been previously explored in the literature. Despite the rarity of these variants, our findings contribute valuable insights and help fill knowledge gaps regarding the clinical management and safety considerations for this specific patient population.

In conclusion, our study suggests that a more conservative *DPYD*-guided dose adjustment could enhance tolerance and decrease the likelihood of treatment discontinuation. This approach could potentially enable a better selection of patients intolerant to slightly higher doses, allowing them to complete treatment. However, further evidence is needed from larger studies in homogeneous populations.

## Data Availability

The original contributions presented in the study are included in the article/Supplementary Material, further inquiries can be directed to the corresponding authors.
