# Supplementary material for: DPYD-guided fluoropyrimidine dose adjustment in colorectal cancer DPYD carriers: start slower to finish stronger
Source: Front Pharmacol. 2025 Sep 18;16:1645188. doi: 10.3389/fphar.2025.1645188 (PMC12488590; doi:10.3389/fphar.2025.1645188)
Supplement: Supplementary file 1 [file Table1.docx]

Supplementary Material

# Supplementary Table 1

| *DPYD* status | Overall Population (n=60) |
| --- | --- |
| c.1236G>A heterozygous  c.2846A>T heterozygous  *DPYD*2A* heterozygous  *DPYD*13* heterozygous  c.1236G>A and c.2846A>T heterozygous | 40 (66,6%)  12 (20%)  6 (10%)  1 (1,6%)  1 (1,6%) |

**Supplementary Table 1. DPYD mutations detected in the overall group of carriers**

# Supplementary Table 2

|  | DA (n=14) | NDA (n=6) |
| --- | --- | --- |
| **Initial Dose**  Relative dose intensity*, median (min-max)  Dose 50%, n (%)  Dose >50%-75%, n (%)  Dose >75%, n (%) | 50% (50%-50%)  14 (100%)  0 (0%)  0 (0%) | 100% (80%-100%)  0 (0%)  0 (0%)  6 (100%) |
| **Maximum Dose**  Relative dose intensity*, median (min-max)  Dose 50%, n (%)  Dose >50%-75%, n (%)  Dose >75% - <100%, n (%) | 75%; (50%-90%)  1 (7%)  11 (79%)  2 (14%) | 100% (80%-100%)  0 (0%)  0 (0%)  6 (100%) |
| **Final Dose**  Relative dose intensity*, median (min-max)  Dose 0%-50% **, n (%)  Dose >50%-75%, n (%)  Dose >75%, n (%) | 75%; (50%-90%)  1 (7%)  11 (79%)  2 (14%) | 37%; (0%-85%)  3 (50%)  1 (17%)  2 (33%) |

**Supplementary Table 2. Fluoropyrimidine dose adjustment dynamics for decreased function *DPYD* variants according to the DA and NDA cohorts** *Relative dose intensity: the given dose relative to the standard dose.** If the treatment was discontinued, the final dose was considered 0%. FP: Fluoropyrimidines; DA: *DPYD*-guided FP dose adjustment cohort; NDA: No *DPYD*-guided FP dose adjustment cohort
